# Short-term memory advantage for brief durations in human *APOE* ε4 carriers

**DOI:** 10.1038/s41598-020-66114-6

**Published:** 2020-06-11

**Authors:** Nahid Zokaei, John Grogan, Sean James Fallon, Ellie Slavkova, Jonathan Hadida, Sanjay Manohar, Anna Christina Nobre, Masud Husain

**Affiliations:** 1 Oxford Centre for Human Brain Activity, Wellcome Centre for Integrative Neuroimaging, Department of Psychiatry, University of Oxford, Oxford, OX3 7JX UK; 2 Department of Experimental Psychology, University of Oxford, Oxford, OX1 3UD UK; 3 Nuffield Department of Clinical Neurosciences, University of Oxford, Oxford, OX3 9DU UK; 4 National Institute for Health Research Bristol Biomedical Research Centre, University Hospitals Bristol NHS foundation Trust and University of Bristol, Oxford, UK; 5 Oxford NIHR Biomedical Research Centre, Oxford, UK

**Keywords:** Human behaviour, Alzheimer's disease

## Abstract

The Apolipoprotein-E (*APOE*) *ε4* gene allele, the highest known genetic risk factor for Alzheimer’s disease, has paradoxically been well preserved in the human population. One possible explanation offered by evolutionary biology for survival of deleterious genes is antagonistic pleiotropy. This theory proposes that such genetic variants might confer an advantage, even earlier in life when humans are also reproductively fit. The results of some small-cohort studies have raised the possibility of such a pleiotropic effect for the *ε4* allele in short-term memory (STM) but the findings have been inconsistent. Here, we tested STM performance in a large cohort of individuals (*N* = 1277); nine hundred and fifty-nine of which included carrier and non-carriers of the *APOE ε4* gene, those at highest risk of developing Alzheimer’s disease. We first confirm that this task is sensitive to subtle deterioration in memory performance across ageing. Importantly, individuals carrying the *APOE ε4* gene actually exhibited a significant memory advantage across all ages, specifically for brief retention periods but crucially not for longer durations. Together, these findings present the strongest evidence to date for a gene having an antagonistic pleiotropy effect on human cognitive function across a wide age range, and hence provide an explanation for the survival of the APOE *ε4* allele in the gene pool.

## Introduction

The Apolipoprotein-E (*APOE*) *ε4* gene allele is the highest known genetic risk factor for developing Alzheimer’s disease (AD)^1^. Approximately 45% of carriers of the gene develop AD by the age of 85 years, compared to 10% of non-carriers^1^. It is not surprising therefore that much research has focused on seeking to identify early biomarkers related to the development of AD in *ε4* carriers^2–13^. But why has this genetic allele, which has such obvious detrimental effects in old-age, been preserved in the human population world-wide?

One possible explanation is rooted in a concept that has emerged in evolutionary biology^14^. The antagonistic pleiotropy hypothesis proposes that some genetic alleles have different effects on the fitness of an organism at different ages. Therefore, a genetic allele, such as *APOE ε4*, which confers a disadvantage later in life, might instead provide an advantage, even earlier in life, hence ensuring its survival. Because the power of natural selection to disfavour a genetic allele wanes later in reproductive life, when an animal has less likelihood of passing on its genes, disadvantages in older age have minimal consequences on the survival of a genetic variant.

Although there has been little investigation of a potential early advantage in human carriers of the *APOE ε4* gene, several authors have argued for the existence of such an advantage, specifically when it comes to brain function^15–17^. A few studies on small cohorts have presented mixed evidence for a possible cognitive advantage in young and middle-aged *APOE ε4* gene carriers. In children and young adults, better performance on neuropsychological measures of attention, executive functioning and even episodic memory encoding^18–22^ have been reported in individuals with the *ε4* allele. In middle-aged adults, while some investigators have demonstrated superior short-term memory (STM) performance in *ε4* carriers^23–25^, others have failed to find such an advantage or report instead worse cognitive function in this group^26–30^.

One major drawback of studies to date, likely contributing to inconsistent results, is the very small sample sizes employed by most investigations in this field (*N* = 60–100). Moreover, previous studies have targeted specific age ranges with no attempt at testing these cognitive functions uniformly across a wide age-range. If *APOE ε4* indeed confers a cognitive advantage, this should be observable across age groups, including in elderly *ε4* carriers, who are of course known to be at greatest risk of developing AD. However, strong evidence for this does not currently exist.

To address these shortcomings and to investigate the existence of a potential cognitive advantage associated with *APOE ε4* gene across ageing, we examined the largest cohort of genotyped individuals to be tested on a highly sensitive test of STM. The task, previously shown to detect subtle changes in performance in healthy ageing and in APOE *ε4* gene carriers in small sample sizes, is a more sensitive measure of memory compared to classical neuropsychological assessments^31^ and also allows separating out the different kinds of error that people make^32,33^.

The current study provides the first evidence in a large sample of participants for a memory advantage over brief durations in *APOE ε4* carriers, across ageing. In addition to providing support for the antagonistic pleiotropy hypothesis, this investigation documents changes in STM that occur with healthy ageing, and thereby provides a comprehensive profile of STM performance in *APOE ε4* gene carriers and non-carriers, on a large-scale, that will aid in delineating cognitive markers associated with AD-related pathology, compared to non-AD related effects of this gene.

Overall, 1277 participants (age range: 20–81), completed the Oxford Memory Test (OMT), a tablet-based task which allowed for remote on-line testing of STM (see Table 1 for demographic information and Methods section for a more detailed description of the participants).

**Table 1:**
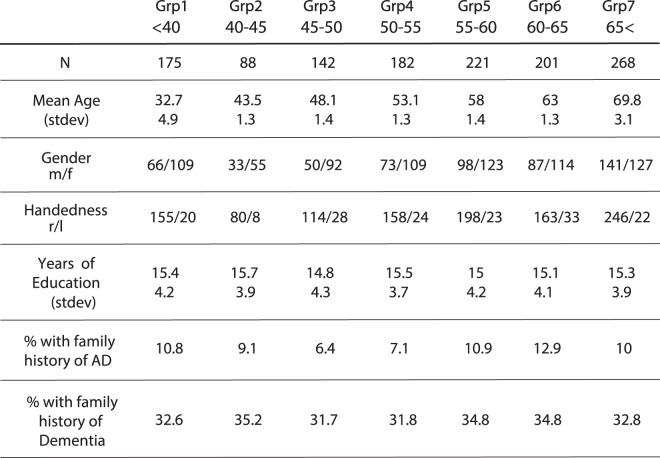
Demographic characteristics of participants across ageing.

**Figure 1:**
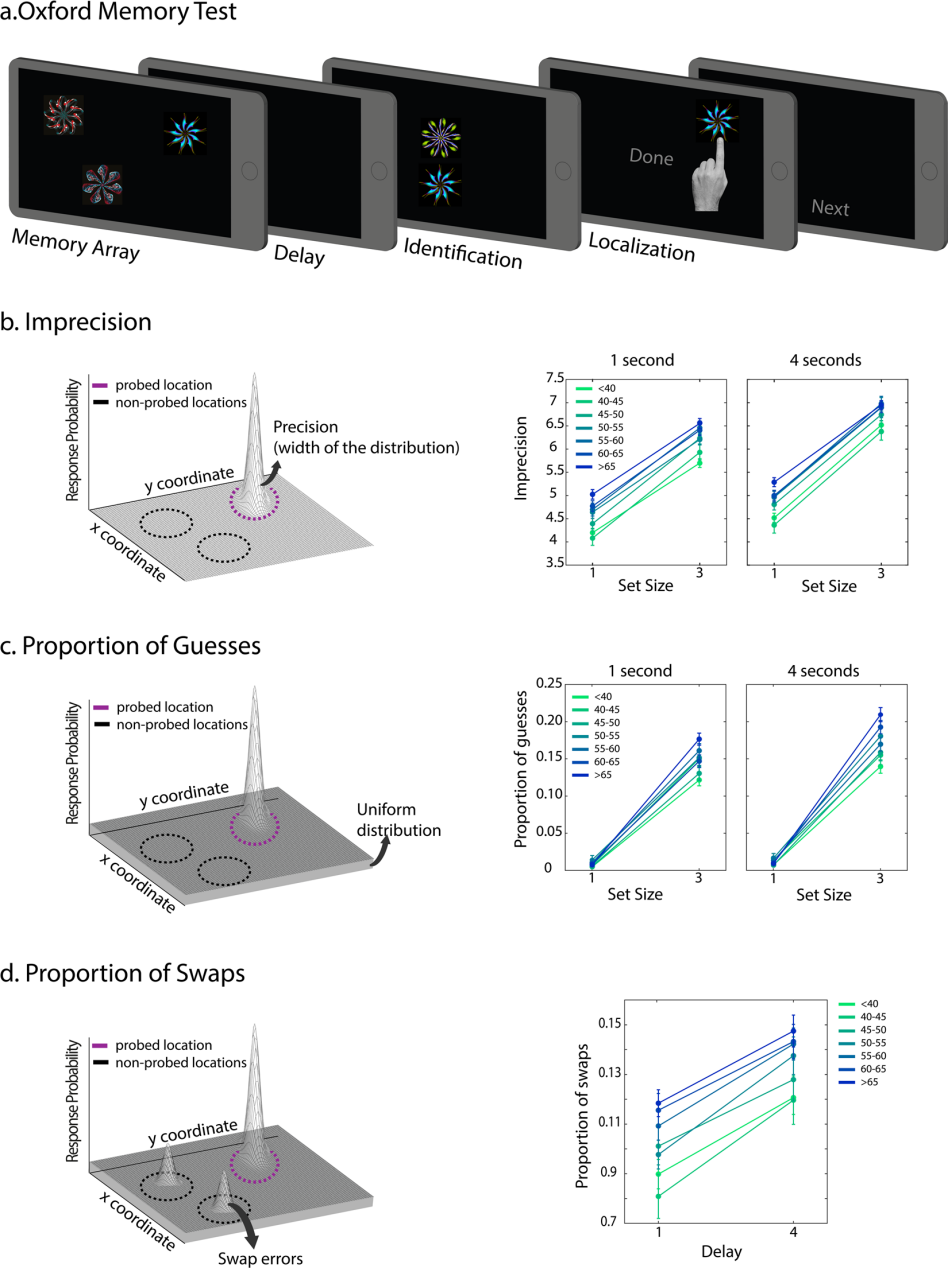
OMT task and sources of error contributing to STM task performance across ageing. (**a**) Schematic of the short-term memory task delivered by the OMT app. (**a**) Localization imprecision is measured as the concentration of a 2-dimentional multivariate Gaussian distribution. Larger values correspond to lower localization resolution. Older participants have lower memory resolution regardless of memory set size or delay. (**b**) Proportion of guesses are proportion of trials in which participants are placing the fractals at a random location. This is captured by a uniform distribution across the screen. Older participants in larger set sizes made significantly more of these types of errors. (**c**) Proportion of swaps. Swap error occur in trials in which participants place the fractal close to one of the other non-probed fractal positions from the memory array. Older participants were making more of swap errors, regardless of delay duration.

A schematic of the task is presented in Fig. 1a. In short, in each trial of the task, participants were presented with a memory array, consisting of either 1 or 3 fractal objects followed by a short or long delay: 1 or 4 seconds. Participants had to keep in mind both the identity and location of the fractals. Following the delay, two fractals appeared at the centre of the screen, one from the original memory array (target fractal) and a novel fractal (foil). Participants first picked the fractal they recalled seeing in the memory array (identification performance) and then dragged it to its remembered location (localization performance). The OMT is a tablet task based on an identical task previously used to examine STM performance in carrier and non-carriers of the *APOE ε4* ^24,34^.

### Short-term memory impairment as a function of age

First, we examined the effect of age on STM performance using the OMT test and mixture modelling of error in 2-dimensional space (see Methods) to quantify the different types of error committed as a function of age. Mixed ANOVAs with the number of objects in the memory set and the duration of the delay as within-subject factors and age-group as between-subject factors were conducted. Both identification accuracy and localization error were influenced by memory set size, delay duration and their interaction between the two factors (see Table S1 for complete statistics). Importantly, older participants performed significantly worse than younger individuals for both identification accuracy (*F*(6,1270) = 9.9, *p* < 0.001, η^2^_p_ = 0.05) and localization error (*F*(6,1270) = 13.7, *p* < 0.001, η^2^_p_ = 0.06).

This occurred specifically for larger memory set sizes (significant interaction between age and set size for identification accuracy: *F*(6,1270) = 10.9, *p* < 0.001, η^2^_p_ = 0.05 and for error: *F*(6,1270) = 8.7, *p* < 0.001, η^2^_p_ = 0.04). Follow-up analyses revealed that older participants were significantly more imprecise for both set size 1 (*F*(6,1270) = 5.7, *p* < 0.001, η^2^_p_ = 0.03) and set size 3 (*F*(6,1270) = 12.9, *p* < 0.001, η^2^_p_ = 0.06); whereas identification accuracy was only impaired in memory set size of 3 (*F*(6,1270) = 11.9, *p* < 0.001, η^2^_p_ = 0.05).

Importantly, when gender of the participants was added as a between-subject factor to all of the analysis conducted above, there was no change in the direction of the findings, with significant interaction between age group and memory set size for both identification accuracy and localization error (see Table S2 for complete statistics).

These findings support previous studies in smaller samples^35,36^, demonstrating an impairment of STM across ageing, with deficits in performance even when only one location had to be retained, illustrating the sensitivity of localization error as a metric. Note that the degradation of STM performance in older participants was modulated by memory set size alone and not delay duration.

To further the sources of error contributing to impaired memory performance in ageing and its modulation by memory set size, we next applied a statistical mixture model of response error to each participant for each set size and delay conditions (see Analysis for more detailed description of the model). According to this model, error can arise from imprecise memory for object locations (increased noise), from random responses (guesses or complete corruption of memory as con occur with attention lapses), or from swap errors (in set size 3 only). Swap (a.k.a. misbinding) errors occur specifically on trials in which participants pick the correct fractal but place it in the location of one of the other items from the memory array. This model has been extensively applied to one dimensional features such as orientation, motion or colour^32,37–39^. Here, we applied an extension of this model to the two-dimensional feature of spatial location^33^.

Model estimates of imprecision, proportion of guesses and swaps were all modulated by age. Firstly, older participants were less precise in recalling spatial locations compared to younger individuals (*F*(6,1270) = 7.9, *p* < 0.001, η^2^_p_ = 0.04), regardless of set size or delay (see Table S1 for all summary statistics), even when one location had to be recalled for the short delay period of 1 second (Fig. 1b). Secondly, older participants made significantly more guesses (*F*(6,1270) = 5.6, *p* < 0.001, η^2^_p_ = 0.03), which was also modulated by memory set size, as observed by a significant interaction between set size and age group (*F*(6,1270) = 7.8, *p* < 0.001, η^2^_p_ = 0.04, Fig. 1c). Follow-up analysis per set size demonstrated a significant effect of age for set size 3 only (*F*(6,1270) = 7.5, *p* < 0.001, η^2^_p_ = 0.03) with no effect of delay. Thus, age influenced the proportion of guesses only in trials in which more than 1 item had to be retained in memory. Lastly, older participants made significantly more swap errors (*F*(6,1270) = 5.2, *p* < 0.001, η^2^_p_ = 0.02), though this was not influenced by the duration of the retention period. Importantly, when gender of the participants was added as a between-subject factor to all of the analysis conducted above, there was no change in the direction of the findings (see Table S3 for complete statistics).

Overall, these findings show that the degradation of STM performance in older participants was most prominent in conditions with higher memory load, i.e., larger memory set size, but with no effect of memory retention period. The increased impairment with ageing was associated with an increase in proportion of both guesses and swap errors.

Memory is well established as a core cognitive function that is greatly affected by healthy ageing^35,36,40–44^. The current findings presented here extend previous reports by demonstrating changes in STM performance in ageing with finer granularity and using a remote test of memory performed by previously genotyped individuals. The degradation in STM performance was attributed to an increase in imprecision in older adults, even for keeping in mind a single object location. More prominent deficits in STM were observed in older participants with higher memory load. This impairment was associated with an increase in proportion of guesses as well as an in swap errors. The latter is indeed consistent with previous reports^42,43,45^. However, the extent to which swap errors characterise STM impairments in ageing is debated as some investigations have found that they account for only a small proportion of age-related variance^41^. In this study, we found a small, but significant, increase in swap errors with ageing.

Additionally, for larger set sizes, age-related STM deficit was accompanied by an increase in proportion of random responses or guesses. This potentially points to a possible impairment in encoding or an increase in attentional lapses in older participants when they have to remember more than one item. Because there was no significant interaction between proportion of guesses and memory delay, any change in attentional lapses during the retention interval is unlikely to be an important contributory factor. In contrast, encoding deficits in older participants have been previously reported by Noack and colleagues^46^. Notably, their study did not model swap errors, attributing to guesses all trials in which the object was placed away from the target location. Here we show that, even when swap errors are taken into account, there is evidence of increased guessing in healthy normal ageing.

### Advantage for brief memory duration in APOE ε4 carriers

Of the full group of participants, 554 had the *ε3/ε3 APOE* variant while 405 were *ε4* carriers (including both *ε3/ε4 and ε4/ε4* carriers). Heterozygous and homozygous *ε4* carriers were treated as one group due to small number of participants in the homozygous group (46 participants across the ages). Table 2 is a summary of demographic information for the two groups. In these two groups, we therefore could examine the influence of the *APOE ε4* gene allele on STM performance by comparing performance in the OMT task across ageing.

**Table 2:**
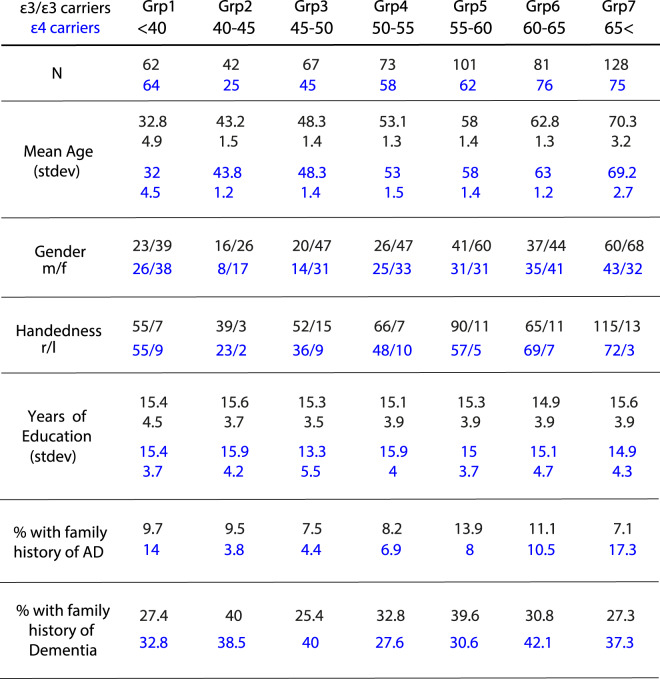
Demographic characteristics of *ε3/ε3 (black) and ε4* carriers (blue).

**Figure 2:**
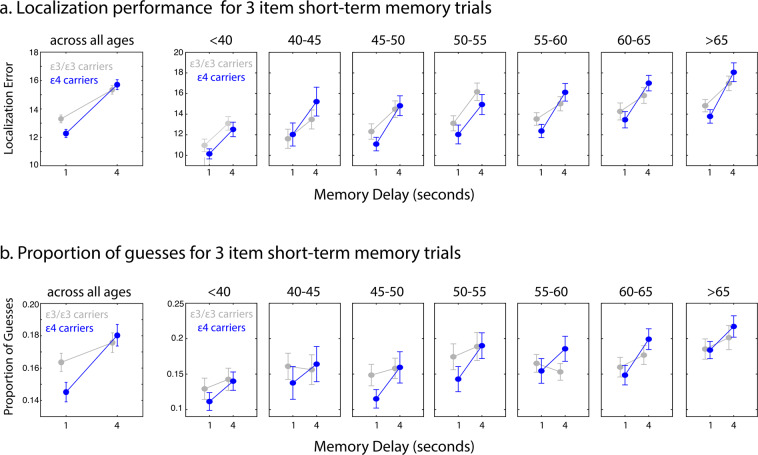
STM task performance in individuals with *ε3/ε3* and *ε4* carriers in the 3 item memory trials. (**a**) *ε4* carriers performed better in the 3 item 1 second condition compared to *ε3/ε3* non-carriers (let panel). Interestingly however, the cost in performance was higher in these individuals following a 4 second delay (right panel). (**b**) The advantageous STM performance and the rapid forgetting in *ε4* carriers, as compared to individuals with *ε3/ε3*, is explained by modulation in proportion of guesses by the *APOE* gene.

Mixed ANOVAs with the number of objects in the memory set and the duration of the delay as within-subject factors and age-group and *APOE* gene status as between-subject factors were conducted. For the entire group, across age, *APOE* status interacted significantly with memory set size and delay for localization performance (*F*(3, 945) = 7.3, *p* = 0.007, η^2^_p_ = 0.01). This 3-way interaction was followed up by further 2-way analysis per set size. For set size 3, there was a significant interaction between delay and *APOE* status (*F*(1,945) = 7.6, *p* = 0.006, η^2^_p_ = 0.08), with *ε4* carriers performing better than non-carriers following a 1-second delay (main effect of *APOE* status: *F*(1,945) = 3.7, *p* = 0.05, η^2^_p_ = 0.004) but with no effect of APOE status following the longer delay of 4 seconds (Fig. 2a) or for memory set size 1.

Thus, *ε4* carriers demonstrated superior STM performance following the short delay period of 1-second with the larger memory set size, that is in the more difficult high load STM condition. Intriguingly, as observed by the interaction between *APOE* status and delay, *ε4* carriers demonstrated rapid forgetting from 1 to 4 seconds, such that that these individuals started with better memory performance for the 1 second delay but performed similarly to non-carriers after a 4 second period (Fig. 2a). This effect did not differ significantly across age groups.

*APOE* status also interacted significantly with memory delay (*F*(1,945) = 4.03, *p* = 0.038, η^2^_p_ = 0.005) for identification accuracy. *APOE ε4* carriers performed better than non-carriers after the short delay of 1 second; and worst after a 4 second delay (though follow-up analysis yielded no further significant effects here; see Table S2 for complete statistics).

What might be the source of such advantageous memory performance to start with and the later accelerated decay? In order to understand the sources of error modulated by the *APOE ε4* gene allele, we next examined model estimates in *ε3/ε3* and *ε4* carriers. There was no effect of *APOE* status on model estimates of imprecision or proportion of swap errors (Table S2). However, there was a 3-way interaction among set size, delay and APOE status (*F*(1, 945) = 4.2, *p* = 0.04, η^2^_p_ = 0.004) for proportion of or guesses.

Follow-up analysis revealed that only for memory set size 3, there was a significant interaction between delay and *APOE* status (*F*(1,945) = 4.7, *p* = 0.029, η^2^_p_ = 0.005, Fig. 2b). For the shorter delay of 1 second, there was a significant main effect of allele (*F*(1,945) = 4.3, *p* = 0.04, η^2^_p_ = 0.004), with *ε4* carriers making significantly fewer guesses. There was no difference in proportion of guesses between the two genetic groups, for longer delays of 4 seconds. This effect did not differ significantly across age groups. This pattern of modulation of proportion of guesses by *APOE* status match those observed in localization error, consistent with the possibility that differences in STM performance as a function of *APOE* status results from differences in the frequency of guesses.

Importantly, when gender of the participants was added as a between-subject factor to all of the analysis conducted above, there was no change in the direction of the findings (see Table S4-5 for complete statistics). Gender interacted with *APOE* status, delay, and set size for the identification accuracy. However, follow-up analyses revealed no differences in the pattern of performance between male and female participants. This may be due to the fact that even though our study includes a large cohort of participants, the number of participants can be small per age group and gender (e.g. 8 male participants in middle-aged APOE *ε4* carriers). Nonetheless, none of the main patterns of findings were influenced by adding gender as an additional factor.

In summary, regardless of age, carriers of the *APOE ε4* gene, that is individuals at a higher risk of developing AD, paradoxically demonstrated a STM memory advantage, specifically for shorter retention periods of 1 second. This pattern of performance was accompanied by a decrease in proportion of guesses made by these individuals. These findings provide the strong evidence for an advantage associated with the *ε4* allele in humans, in line with the broader antagonistic pleiotropy hypothesis. In the same group of individuals, there appeared to be an increase rate of rapid forgetting. In other words, there was a larger cost in memory performance following longer delays of just 4 seconds in memory. To the best of our knowledge, these findings present the strongest evidence to date for a gene having an antagonistic pleiotropy effect on human cognitive function across a wide age range. They also provide an explanation for the survival of the *APOE ε4* allele in the gene pool. Until very recently, most people did not live to an age where they would develop Alzheimer’s disease. Rather only the advantages would have been apparent, which we now document to be present across all ages.

The results complement and extend and strengthen observations in far smaller samples of superior STM performance in middle-aged and older participants^24,25,47^. Most importantly, they show that the effect is consistent across a wide age group, and hence distinguishable from AD-related effects of this gene on cognition. Previously, it has been suggested that better cognitive performance in such at risk groups may arise due to increased (or compensatory) recruitment of regions that are not directly linked to AD pathology, for example frontal and parietal regions due to ‘phenotypical effects’ of this gene – that is non-AD related effects of the gene on cognition. Consistent with such a proposal, several studies have reported increased activity in these regions in carriers of the *APOE ε4* gene compared to non-carriers during STM task performance^48–50^. Given the established role of these brain regions in STM and attentional processes^51–56^, it might be speculated that in ε4 gene carriers greater engagement of these areas results in better memory performance for very short delays.

The benefit in memory for brief duration was exclusively explained by a reduction in number of guesses carriers made compared to non-carriers, as estimated by computational modelling of spatial error. This additional methodological approach may shed some light on the underlying cognitive processes associated with such a memory advantage, which in turn potentially leads to a possible functional and evolutionary benefit. A decrease in the number of guesses for brief durations might arise from reduced random corruption of information at any one of the different stages of STM: encoding, maintenance or retrieval. Of relevance, previous studies in small samples have reported encoding benefits^20^ (including on episodic memory tasks^6,18^) or enhanced visual attention^21^ in *ε4* carriers. The evidence provided in the current investigation together with the results of these prior reports, suggest that these individuals are better at rapid processing of visual information, holding on to recently attended information while re-directing their focus to the next relevant target. The advantage is unlikely to be simply due to improved visual processing, because the advantage was not seen when single items had to be remembered and importantly, was delay-dependent. This specific cognitive benefit may provide a functional advantage in any task that requires rapid computations such as foraging, visual search, decision-making or when exploring new environments, highlighting the potential evolutionary benefit associated with the *APOE ε4* allele.

Contrary to this advantage for brief durations, carriers of the *APOE* ε4 gene exhibited a greater rate of forgetting as demonstrated by comparable performance between the two groups following longer memory delays of only 4 seconds. Previously, studies have reported increased forgetting in middle-aged and older *APOE* ε4 carriers on long-term memory tasks^4,5,9,26,28,34,57–59^. Here, we demonstrate an increase in rapid forgetting in these individuals at much faster time scales, in a matter of seconds, in line with the possible prodromal effect of the APOE gene on longer-term memory. This distinction between very brief and longer durations is only possible by using a computerised test of STM that manipulates retention intervals.

The data presented here also demonstrates that it is possible to use a sensitive and easy-to-administer test of STM remotely to obtain data at large scale. The task proved to both sensitive to age-related changes in STM performance and variations in the genetic make-up of participants. This is a critical step forward in providing a fingerprint of cognition that might potentially be helpful when identifying and separating early signs of AD from non-AD related effects of the ε4 allele. Lastly and importantly, the results provide normative data which can be used as a benchmark for future investigations with smaller sample sizes and clinical populations.

## Methods

Experimental procedures were reviewed and approved by the Central University Research Ethics Committee of the University of Oxford. The experiment was conducted in accordance with their policy on research involving human participants and personal data.

### Participants

Overall 1277 participants were recruited through the NIHR BioResources (https://bioresource.nihr.ac.uk/) and completed the study remotely. Upon agreeing to take part, each participant was sent a unique ID number and a step-by-step guide on how to complete the study. Participants firstly had to complete an online consent form, approved by the University of Oxford Ethics Committee. This was followed by a set of demographics questionnaires administered online. Lastly, participants downloaded and performed the Oxford Memory Task app (OMT) on their tablets, at a time suitable to their schedule. See Table 1 for demographics of participants. There was no significant difference in years of education and proportion of participants reporting a family history of AD or dementia. Family history of AD or dementia was calculated based on reported cases of AD or dementia in parents or grandparents of the participants. It’s important to note though that these may not reflect a confirmed clinical diagnosis.

Genetic information regarding the participants’ *APOE* allelic variants was provided after data collection was completed by the NIHR BioResources (for APOE genotyping methods please refer to the NIHR BioResrouces website: https://bioresource.nihr.ac.uk/). Considering that the focus of this study is to extend previous studies on the advantageous short-term memory performance in individuals at risk of developing AD, heterozygous and homozygous carriers of the *APOE* ε2 gene were excluded from further analysis. From the full aging group, five hundred fifty-four had the *ε3/ε3 APOE* variant whil3e four hundred and five were *ε4* carriers (including both *ε3/ε4 and ε4/ε4* carriers – see Table 2 for demographic information for the two groups). There was no significant difference in years of education, gender, and handedness distribution, or proportion of participants reporting a family history of AD or dementia between the two groups.

### Oxford memory test (OMT)

The Oxford Memory Test (OMT) web-app (https://omt.psy.ox.ac.uk/app/omt/#login) is a flexible platform that is designed for testing in ‘less strict’ environments, in particular for use in clinics and wards. The test is patient friendly. It is administered on a touchscreen and can run on any tablet or touchscreen device, as well as in a browser from a desktop computer. Moreover, the test can be performed offline; all the data is stored on the device and transmitted securely to the server when connected to internet. Further, the stimuli adapt to different screen sizes and resolutions, by either inputting the device model or the specific resolution while assuming a constant viewing distance.

The OMT platform is designed in a way that can be programmed to use arbitrary stimuli, presented at any specified size. The short-term memory task specifics can also be easily manipulated. For example, it is possible to change the retention duration delay or the nature of presentation of the memory stimuli (e.g. simultaneously or sequentially). Such flexibility allows testing of any specific mechanistic hypotheses.

For the current study however, we focused on a version of the task that has been previously used in our lab, administered on a touchscreen computer, and has been successful in detecting subtle changes in performance in healthy ageing, neurodegenerative disorders and in at-risk populations^24,36,60,61^. A schematic of the task is presented in Fig. 1a.

In summary, in each trial, participants were presented with 1 or 3 coloured fractals (memory array) for 1 or 3 seconds respectively. The memory array was followed by either 1 or 4 seconds black blank display. This was followed by the recall phase. At recall, participants were presented with 2 fractals centrally along the vertical meridian; one from the memory array and one foil (i.e. a fractal that did not appear in the memory array). Participants had to select the fractal that appeared in the memory array (identification step) and drag it to its remembered location (localization step). To confirm the final response, participants had to press the “Done” button at screen centre that appears only following the identification step. This was followed by an empty screen with a “Next” button at the lower edge of the screen, at centre.

The stimuli were randomly selected from a pool of 196 coloured fractals, with maximal width and height of 3° of visual angle. The location of each fractal was determined in a random manner, with few restrictions. Fractals had a minimum distance of 4° of visual angle from each other, a minimum distance of 1.5° from the edges of the screen and a minimum distance of 2° from screen centre, assuming a viewing distance of 40 cm.

Participants completed 2 blocks of 40 trials, each block consisted of 10 trials per condition. Prior to the beginning of the task, participants were acquainted with the experimental design and conditions by firstly completing 2 trials, with written instructions on each frame, and a further 8 trials of practice trials with no instructions. Once these trials were completed, participants started the experimental blocks and were told to take a break in between the blocks.

### Analysis

#### Behavioural analysis

For identification accuracy we measured the proportion of trials in which participants correctly identified the fractal. For localization error, coordinates of fractals from the memory array and the response coordinates were normalized by screen dimensions to lie between 0 and 100 (as participants used different size screens). Localization error was calculated as the distance between the reported and true location of the probed item (Euclidean distance).

#### Mixture modelling of error

To identify sources of error associated with ageing or the *APOE* gene, a model with three sources of error was applied to localization step of the task. According to this model, error can arise to either imprecision (Fig. 1b- left panel), random responses due to guesses (Fig. 1c- left panel) or swap errors (Fig. 1d- left panel). Swap errors arise in trials in which participants pick the correct fractal but place it in the place of one of the “other” non-probed items from the memory array. This model has been so far very successful in identifying sources of error in memory tasks that use reproduction of one dimensional features such as orientation, colour or motion^32,37^. Recently, this has been adapted for 2 dimensional features such as location in the MemToolbox 2D package^33^. The model is descried by the following equation:

$$P(\hat{\theta })=\alpha {\psi }_{K}(\hat{\theta }-\theta )+\beta \frac{1}{M}{\sum }_{i}^{m}{\psi }_{K}(\hat{\theta }-{\varphi }_{i})+\gamma \frac{1}{A}$$

where the free parameters of α, β, γ, and κ, correspond to proportion of target responses, swaps, guesses and the concentration of a 2D bivariate Gaussian distribution (with zero covariance) respectively. The $\hat{\theta }$ parameter corresponds to the coordinates of the response, $\theta$ to the target coordinates, ${\varphi }_{i}$ to the coordinates of non-probed item *i*, $\psi$ to the bivariate Gaussian distribution, and *A* to the screen dimensions.

The model was fit to each trial type separately (excluding practice trials) for the participants, and was fit using maximum likelihoods^62^. Normalised localization error was used to estimate model parameters, as described above, thus the concentration parameter is in units of ‘% of screen size’.

For both behavioural outcomes as well as model estimates, mixed ANOVAs were performed with set size and delay as within-subject factors and age group and/or *APOE* status as between-subject factors.

## Supplementary information

Supplementary Information.
