# Supplementary material for: Short-term memory advantage for brief durations in human APOE ε4 carriers
Source: Sci Rep. 2020 Jun 11;10:9503. doi: 10.1038/s41598-020-66114-6 (PMC7289888; doi:10.1038/s41598-020-66114-6)
Supplement: Supplementary file 1 — Supplementary Information. [file 41598_2020_66114_MOESM1_ESM.docx]

Short-term memory advantage for brief durations in human *APOE* ε4 carriers

Nahid Zokaei^1,2,a^, John Grogan^3^, Sean James Fallon^4^, Ellie Slavkova^2^, Jonathan Hadida^1^, Sanjay Manohar^3^, Anna Christina Nobre^1,2, †^ & Masud Husain^2,3,5, †^

† Denotes equal contribution

1. Oxford Centre for Human Brain Activity, Wellcome Centre for Integrative Neuroimaging, Department of Psychiatry, University of Oxford, Oxford, UK, OX3 7JX
2. Department of Experimental Psychology, University of Oxford, Oxford, UK, OX1 3UD
3. Nuffield Department of Clinical Neurosciences, University of Oxford, Oxford, UK, OX3 9DU
4. National Institute for Health Research Bristol Biomedical Research Centre, University Hospitals Bristol NHS foundation Trust and University of Bristol
5. Oxford NIHR Biomedical Research Centre

**Corresponding author**

^a^To whom correspondence should be addressed at:

Centre for Human Brain Activity (OHBA)

Department of Psychiatry

University of Oxford

Oxford

OX3 7JX

United Kingdom

Email: [nahid.zokaei@psy.ox.ac.uk](mailto:nahid.zokaei@psych.ox.ac.uk)

**Supplementary material**

**Table S1**. Summary statistic on performance in the STM task across ageing. For each outcome measure, significant interactions were followed up by further tests, presented in a separate section.

**Table S2** Summary statistics on performance in the STM task across ageing with gender of participants as an additional between-subject factor.

**Table S3** Summary statistics on sources of error in the STM task across ageing with gender of participants as an additional between-subject factor.

**Table S4**. Summary statistics on performance in the STM task across ageing for carrier and non-carriers of the *APOE* *ε4 gene allele*. For each outcome measure, significant interactions were followed up by further tests, presented in a separate section.

**Table S5**. Summary statistics on performance in the STM task across ageing for carrier and non-carriers of the *APOE* *ε4 gene allele* with gender of participants as an additional between-subject factor.

**Table S6**. Summary statistics on sources of error in the STM task across ageing for carrier and non-carriers of the *APOE* *ε4 gene allele* with gender of participants as an additional between-subject factor.
